# A Systematic Review of Intervention Trials Utilizing Biomarkers Among Informal Caregivers of People with Alzheimer's Disease & Related Dementias

**DOI:** 10.14336/AD.2024.0115

**Published:** 2023-12-23

**Authors:** Adam O’Riordan, Michelle A. Chen, Valentina Maza, Nyla Vela, Lydia Wu-Chung, Alexandria Henderson, Olivia L. Carney, Angie S. LeRoy

**Affiliations:** ^1^Department of Psychology & Neuroscience, Baylor University, Waco, TX 76798, USA.; ^2^Institute for Policy Research, Northwestern University, Chicago, USA.; ^3^Department of Psychological Sciences, Rice University, Houston, TX, USA.; ^4^Department of Biology, Baylor University, Waco, TX 76798, USA.

**Keywords:** Alzheimer’s Disease & Related Dementias;, systematic review, caregiver interventions, ADRD caregivers, biomarkers, biobehavioral interventions

## Abstract

Informal caregivers of people with Alzheimer's Disease and Related Dementias (ADRD) experience unique stressors, reduced quality of life, and report poorer health, compared to non-caregivers. Throughout the last thirty years, researchers have developed and tested various psychosocial interventions and their ability to improve caregiver health. Due to an exclusive focus on self-report methods, however, no existing systematic literature reviews specifically examine intervention studies employing biomarkers; this systematic review aims to address this gap in the literature. In each database (PubMed and Web of Science, respectively), a title search was conducted with the following keywords: “alzheimer*” OR “dementia” AND “caregiv*” AND “intervention”, followed by a second search using identical keywords except “intervention” was replaced with “program.” Study or intervention protocol articles, exclusively qualitative studies, cultural applicability papers, dissemination studies, descriptive articles or program reports, acceptability/feasibility studies, studies utilizing formal caregiving samples, commentaries, review papers, and meta-analyses, erratums/corrections, measure development articles, factor analyses, and case reports were excluded from the final pool of studies. In this systematic review, the findings of 14 studies are summarized, and are organized based on specific types of biomarkers: neuroendocrine, immune, and autonomic physiological. Overall, the review yielded mixed results, which may, in part, be due to differences in the types of interventions tested, as well as differing biomarker measurement, methodology, and analysis. More biobehavioral intervention trials are needed among ADRD caregivers. Including biological parameters as pre- and post-measures can shed insight into the extent to which interventions may help caregivers heal from the stress of caregiving.

## 1. INTRODUCTION

People with Alzheimer's Disease and Related Dementias (ADRD) require continuous attention and care, which intensifies as the disease progresses. For example, primary caregivers of people with ADRD spend over 27 hours (on average) per week providing care, even increasing to upwards of 64 hours per week in the final 12 months of the care recipient’s life [1]. Furthermore, people with ADRD may live with ADRD for a long time (3.3 to 11.7 years), accumulating considerable stress and caregiver burden for their caregivers [2, 3]. Informal caregivers of people with ADRD provide over 16 billion hours of unpaid care, equating to $271.6 billion in 2021 alone [1]. Informal caregivers of people with ADRD experience unique stressors and reduced quality of life, compared to non-caregivers; dementia caregivers are also more likely than non-caregivers or other caregivers to report poor health [4, 5].

Researchers and health providers have strategized ways to support family caregivers of people with ADRD by creating interventions to improve their health and well-being. For example, in the last thirty years, researchers developed and tested various psychosocial interventions (e.g., therapeutic, psychoeducational, social support) and their ability (if any) to improve caregiver health [6-8]. Due to an exclusive focus on self-report methods, existing literature reviews focus on intervention studies that utilize only psychological outcomes [6,9]. None, however, specifically examine intervention studies employing biomarkers. Thus, our review aimed to address this critical gap in the existing literature.

### 1.1 The Psychobiological Burden of Caregiving

Chronic stress among caregivers impacts health through different biobehavioral pathways. Specifically, stress associated with ADRD spousal caregiving impacts health through neuroendocrine pathways, such as the hypothalamus-pituitary-adrenal (HPA) axis. Under normal conditions, cortisol, a stress hormone (i.e., glucocorticoid hormone) released by the HPA axis and regulated by adrenocorticotropic hormone (ACTH), acts to dampen the body's inflammatory response. In contrast, chronically high cortisol levels can desensitize the immune system to the effects of cortisol and impair the body’s ability to regulate inflammation, which increases disease risk [10]. Caregivers exhibit significantly higher levels of ACTH, the hormone that controls cortisol production, than non-caregiver controls. Moreover, caregivers of people with ADRD who provide extended care with little-to-no help exhibit elevated ACTH compared to caregivers under less stressful conditions [11]. Furthermore, spousal caregivers of people with ADRD have higher cortisol levels than healthy controls [11-13]. Caregivers of people who exhibit more behavioral and psychological dementia symptoms also exhibit elevated cortisol responses compared to caregivers of people with less behavioral and psychological dementia symptoms [13]. Additionally, compared to healthy controls, spousal caregivers of people with ADRD also display lower anti-glucocorticoid (indexed by dehydroepiandrosterone [DHEA]) levels, which are affected by chronic caregiving-related stress [12]. The higher ratio between cortisol and DHEA in spousal caregivers of people with ADRD indicates one possible physiological mechanism of chronic stress and its associated mental and physical health outcomes among caregivers.

Chronic inflammation is a key biological mechanism contributing to disease, disability, and death [14]. While the local inflammatory response protects the body from foreign bacteria or viruses, prolonged stress precipitates elevated systemic inflammation, leading to cardiovascular disease and other chronic illnesses [14, 15]. Chronic stress associated with caring for a loved one with ADRD can promote a systemic pro-inflammatory state, increasing susceptibility to diseases in older adulthood [16]. Caregivers of people with ADRD tend to be older adults who are already experiencing age-related declines in immunocompetence [17]; inflammation associated with caregiving further magnifies age-related increases in pro-inflammatory cytokines [16]. Cytokines can act as molecular signals of illness [18], stimulating pathways that are associated with a coordinated set of adaptive behavioral changes seen in both animals and humans, including (but not limited to) lethargy, depressed mood, reduced social exploration, and reduced grooming or self-care [18]. In addition to contributing to morbidity and mortality risk, heightened inflammation and its associated sickness behaviors (e.g., depressed mood, fatigue) may negatively influence caregivers’ health and quality of life. Specifically, higher levels of pro-inflammatory cytokines, including interleukin 6 (IL-6) and tumor necrosis factor-alpha (TNF-α) predict increased morbidity and mortality and decreased quality of life in older adults [14, 19]. Notably, spousal caregivers of people with ADRD demonstrate four-fold greater IL-6 levels, compared to matched controls [16]. These findings indicate that ADRD spousal caregivers could cross into a high-risk morbidity and mortality category earlier in life (at 75 years of age) than their non-caregiving counterparts, who would not reach high-risk until after age 90 [16]. Notably, more recent research by Roth et al. (2023) has revealed a more complex association between caregiving stress and inflammatory biomarkers by examining latent factors of inflammatory biomarkers rather than parameters in isolation. While caregiving was not associated with a latent “up-regulation” factor, primarily characterized by IL-6 and C-reactive protein (CRP), individuals who transitioned into a caregiving role over a 9-year follow-up period were found to display less of an increase over time in an inhibitory feedback factor (i.e., anti-inflammatory biomarker factor), indexed by Interleukin 2 (IL-2) and Interleukin 10 (IL-10) [20]. Thus, chronic stress associated with caring for someone with ADRD negatively impacts health by dysregulating the immune system and increasing chronic inflammation, thereby prematurely aging the immune response [16, 21].

In addition to the impact on neuroendocrine and immune functioning, chronic stress also negatively impacts health through one's psychophysiology. The autonomic nervous system plays a significant role in disease because of its influence over how humans adjust to stressful situations [22]. Within the autonomic nervous system, sympathetic responses involve energy mobilization or "flight-or-fight" functions, such as increased heart rate, while the parasympathetic system includes "rest-and-digest" functions. Under normal conditions, these two branches function in dynamic balance so the body can adjust appropriately to changing environments and prevent maladaptive responses to stress [22]. However, under conditions of chronic stress, the autonomic nervous system becomes imbalanced; an overactive sympathetic system and an underactive parasympathetic system place excessive energy demands on the body during stress that subsequently increase vulnerability to disease [22]. For example, cancer caregivers display autonomic imbalance compared to controls, suggesting an overactive sympathetic system [23]. Autonomic imbalance likely plagues informal ADRD caregivers much like it does cancer caregivers. For instance, long-term caregivers of spouses living with dementia exhibit higher heart rate and blood pressure compared to non-caregiver controls, indicating that caregivers have higher sympathetic activation than non-caregivers [11]. To date, few studies directly investigate autonomic imbalance among caregivers of people with ADRD. Research should continue to examine autonomic imbalance among caregivers of people with ADRD to better understand the biological pathways mediating the relationship between ADRD informal caregiving and health.

### 1.2 Limited Biobehavioral Scope of Existing AD Informal Caregiving Intervention Studies

Taken together, these research findings highlight health risks to caregivers of people with ADRD and implicate several biobehavioral pathways (e.g., immune, neuroendocrine, and psychophysiological) which may partially explain the association between ADRD informal caregiving and poor health. These pathways are prime targets for ADRD informal caregiving interventions and are key variables to consider when developing and testing ADRD caregiving interventions. Yet, the literature on informal ADRD caregiving intervention studies employing biobehavioral measures is limited, at best. In 2001, Farran and colleagues published a call-to-action in their review paper, requesting interdisciplinary researchers to combine their collaborative efforts to pursue ADRD intervention research, investigating neuroendocrine and immune outcomes [24]. Based on our review, in the two decades since Farran et al. (2001), only fourteen studies followed their recommendations to include biological outcomes in ADRD caregiving intervention study designs. Thus, in this systematic review article, we aim to (1) review the limited existing studies, which assessed the impact of ADRD informal caregiving interventions on neuroendocrine, immune, and psychophysiological parameters and (2) summarize the current state of the literature.

## MATERIALS & METHOD

### 2.1 Search Strategy

Following PRISMA guidelines for systematic reviews, we conducted a systematic literature search to review the methodology and findings of scientific studies testing interventions for informal caregivers of people with ADRD, which utilized biomarkers as objective health indicators pre- and post-intervention. Sources included peer-reviewed articles from PubMed and Web of Science published online between June 1987 and April 2022. We conducted several preliminary searches to identify which combination of search parameters yielded the most optimized search. Our final review consisted of results from four searches. In each database (PubMed and Web of Science, respectively), we conducted one title search with the following keywords: “alzheimer*” OR “dementia” AND “caregiv*” AND “intervention”, followed by a second search using identical keywords, except “intervention” was replaced with “program.” We conducted the last search on March 24, 2022. The last author conducted the search, and then led the team in using Covidence Software to sort the results and check for duplicates [25]. All articles underwent selection twice: once by the last author and once by a co-author or trained research assistant. We reviewed titles, abstracts, and in some cases the complete text, for eligibility. We excluded study or intervention protocol articles, exclusively qualitative studies (e.g., intervention development articles using exclusively focus-group or interview data), cultural applicability papers, dissemination studies, descriptive articles or program reports, acceptability/feasibility studies, studies utilizing formal caregiving samples (e.g., paid nurses), commentaries, review papers, meta-analyses (none of which focused on intervention studies utilizing objective biomarkers), erratums/corrections, measure development articles, factor analyses, and case reports. A final search of PubMed and Web of Science was conducted prior to submission in June 2023 to identify any new published articles.

### 2.2 Data Extraction

Two researchers (primary author and one co-author) extracted the following information from each study: (a) intervention type, (b) intervention dosing, (c) study design (including study arms; control or comparison group(s)), (d) sample characteristics (sample size, gender), (e) how each study defined ADRD caregiver, (f) which biomarkers were assessed and when, (g) results for the biomarkers pre-to-post-intervention. The primary author resolved data extraction discrepancies after careful review of the articles in question.

**Figure 1.**
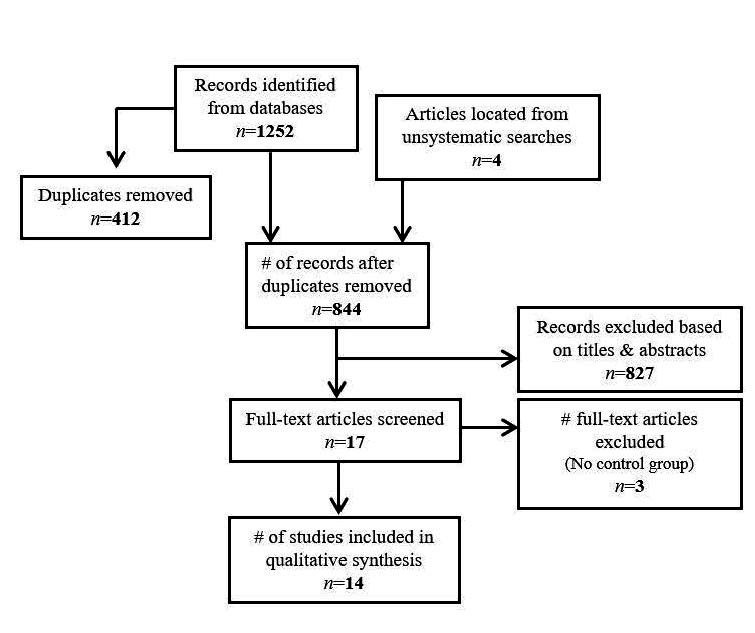
Summary of article screening & eligibility.

## 3. RESULTS

We identified a total of 1252 articles from the databases for screening, 412 of which were duplicates (see Figure 1 for PRISMA diagram). We also added 4 articles to the screening pool, which were identified during previous unsystematic searches of other databases (e.g., Google scholar, PsycINFO), that we noticed were not captured in the PubMed and Web of Science searches. Following the removal of duplicates (412), we screened 844 articles for inclusion. Of the articles reviewed, 17 articles met eligibility criteria. We also searched the reference lists of the 17 selected articles and did not find any new eligible articles to add to the compilation. During the full-text review phase, we noticed that three articles reported studies without control groups, making it difficult to interpret and meaningfully compare the results of these studies to those with a control group [26-28]. Thus, we include only the 14 studies with control groups in the final qualitative synthesis reported below (summarized in Table 1). A final search was conducted prior to submission of this article (June 2023), and no additional studies were identified. We organized the synthesis based on specific types of biomarkers: neuroendocrine markers and neurotransmitters, immune markers, and autonomic physiology indices.

### 3.1 Neuroendocrine Markers & Neurotransmitters

Based on our review, eight ADRD informal caregiving intervention studies included cortisol, a neuroendocrine marker, and two studies included epinephrine and norepinephrine, stress-related neurotransmitters. We will begin by summarizing the articles, which utilized cortisol as a neuroendocrine outcome. Then, we will summarize those which included epinephrine and norepinephrine.

### 3.1.1 Cortisol

Diurnal cortisol refers to the daily cycle of cortisol secretion and is a widely accepted biomarker of emotional and physiological stress reactivity [29-31]. The normative daytime diurnal cortisol cycle shows gradual decline throughout the day with highest levels in the morning [32]. Including cortisol as a neuroendocrine marker in interventions for caregivers of people with ADRD is clinically relevant, given that caregivers exhibit flattened (unhealthy) cortisol slopes [13, 33].

**Table 1:** Study Design, Sample & Intervention Characteristics, & Biomarker Results in ADRD Caregiver Intervention Trials.

Authors (Year)	Total N (% female); *n* for each condition	Trial Arms	Dosing	Biomarkers Included	Measurement timepoints	Results
**Holland et al. (2011)**	125 (100%); *n*=65 CWC *n*=60 TSC	^R^*Coping with Caregiving* (CWC), a psychoeducational, cognitive behavioral intervention or Telephone support control (TSC)^*^	13-16 wks; CWC: small group format, 2 hrs every week; TSC: 15-20 min bi-weekly phone calls from one empathetic support provider	Salivary Cortisol	6 months post-baseline & 2 months after conclusion of intervention; Cortisol: at wake, 5pm, & 9pm across two consecutive days at baseline & post-tx	No tx group differences in waking cortisol levels or diurnal slope. However, there was a tx × intensity of CG situation interaction effect on post-treatment awakening cortisol and diurnal cortisol slopes. High-intensity caregivers in the tx group (CWC), displayed higher wakening cortisol and steeper diurnal cortisol slopes in comparison to high-intensity caregivers in the control group (TSC). TSC (control group, but not CWC) had lower waking cortisol and flatter (less normal) diurnal slopes post-tx compared with those in low-intensity situations.
**Danucalov et al. (2013)**	46 (89%); *n*=25 YCMP *n*=21 Waitlist	^R^Yoga and Compassion Meditation Program (YCMP) or Waitlist control^†^	Across 2 mos (8wks), 3 sessions/wk; 1 live session/wk; 2 (DVD) at-home sessions/wk via; sessions last 1hr 15 min	Salivary Cortisol	Pre- & post-intervention; collected on 2 consecutive days, both right after waking, while fasting, in the dark; second was collected 30 minutes later under exposure to natural light	There was a significant group (YCMP vs. CG) × time (before vs. after intervention) interaction on cortisol levels collected on day two, as well as average cortisol levels collected across the two days. The YCMP Ps showed a reduction in cortisol level on day two (not on day one), and a reduction in the average of day one + day two cortisol levels from pre- to post-intervention. No significant changes were observed amongst Ps in the control group.
**Aboulafia-Brakha et al. (2014)**	27 (81%); *n*=12 CBT *n*=15 EDUC	^SR^Cognitive-Behavioral Group Therapy (CBT) or Psychoeducation Group Program (EDUC) control^*^	CBT: 8 wkly group sessions, 90 mins ea EDUC: 8 wkly group sessions, 60 mins ea	Salivary Cortisol	Pre- & post-intervention; salivary cortisol collected 4x/day: 1) immediately after waking, (2) 30 min after waking, (3) 8 hr after the first sample & (4) immediately before going to sleep	There was a time (pre- vs. post-intervention) × group (CBT vs. EDUC) interactional effect on diurnal cortisol secretion. Significant reductions in daily salivary cortisol secretions were observed only among the CBT group, not the control. The CBT group also displayed a different diurnal cortisol pattern, characterized by attenuated evening cortisol levels post intervention, compared to pre-intervention. High evening cortisol levels remained post-intervention for the control group.
**Brown et al. (2016)**	38 (84%); *n*=23 MBSR *n*=15 SS	^R^MBSR (group-based) or Social Support (SS; group-based) control^*^	8-wks, 1.5-2 hr classes/week; MBSR program also included a day-long intensive mindfulness session	Salivary Cortisol	Pre-, post-, & 3 mo follow-up; salivary cortisol 6x for 1 day: before rising in the morning, 45 min, 2.5 h, 8 h, & 12 h after waking, & bedtime	No treatment condition differences in diurnal cortisol curve pre- to post-intervention or to follow-up points.
**Prick (2017)**	111 (72); *n*=57 Intervention, *n*=54 Control	^R^Physical exercise and support (i.e., psycho-education, communication skills and pleasant activities training), or routine medical care^*^	Intervention: 8 home visits over 3 mos--wkly for 1st mo, then bi-weekly for 8 wks; also prescribed 30 min active exercise ≥ 3 days/wk. Control: (3) monthly info bulletins & (3) monthly phone calls	Salivary Cortisol	Baseline & post-tx cortisol timepoints: right after waking and 30 minutes after waking	No group differences in cortisol from baseline to post-tx for cortisol level at time of morning awakening and cortisol awakening response.
**Waelde et al. (2017)**	31 (100%); *n*= 16 IRMT, *n*= 15 PTS	^R^Inner Resources Meditation and Mantra Training (IRMT) or psychoeducation & telephone support (PTS)^*^	IR: 9 sessions, (8 wkly for 1.5 hrs, + one 3 hr retreat), across 8 wks, + one 1.5 hr booster session at the end of wk 12 PTS: 6 bi-weekly phone calls, 10-15 mins each, over 12 wks	Salivary Cortisol	baseline (1 wk pre-tx) & 1 mo follow-up cortisol timepoints: awakening, 5:00 p.m., and 9:00 p.m	IR participants had flatter cortisol slope than PTS at baseline. IR improved more than the PTS in diurnal cortisol slope. Caregivers in the meditation group demonstrated improved, steeper daytime cortisol slope across the 12 wks of the study relative to controls.
**Kim et al. (2011)**	44 (91%); *n*= 25 experimental *n*= 19 control	Support Program or Control Group^†^	Once wkly sessions (2-3 hrs per session) for 8 wks	Epinephrine (E) & Norepinephrine (NE)	pre- & post-intervention	No significant differences in E from pre-to-post intervention or change in E between groups. NE levels significantly increased from pre- to post- intervention in controls but did not significantly increase for experimental group. Differences between groups in NE changes pre- to post-intervention, with the experimental groups displaying less of an increase in NE.
**Garand et al. (2002)**	37 (92%); Individual group *n*s not reported	^R^Progressively Lowered Stress Threshold (PLST) psychoeducation or Matched Support^*^	Two 3 hr home visits; then, phone call every other wk, for 6 mos	NK cell activity, T-cell proliferation to PHA & ConA	baseline, 1 wk post-intervention, after 6 months of bi-weekly phone contact	No significant group × time interaction on NK cell activity or T-cell proliferation. PLST group showed stronger T-cell proliferation to PHA & ConA, compared to control, after the in-home intervention and after 6 months of telephone support.
**Oken et al. (2010)**	31 (80%); *n*=10 mindfulness meditation *n*=11 education *n*=10 respite	^R^Mindfulness meditation or Education^*^ or Respite Only (pragmatic control)^†^	Mindfulness: 6 wkly 90 min group sessions Education: 6 wkly 90 min lectures Respite only: 3 hours, 1x per wk, for 7 wks	Salivary Cortisol, IL-6, TNF-α, CRP	pre- (3 wks before), post-intervention (3 wks after); salivary cortisol: 3 times during the assessment day (5 mins after waking, 30 mins later before eating, and at bedtime 10-11pm)	No tx effect on salivary cortisol, cytokines, or CRP
**Moore et al. (2013)**	100 (74%); *n*= 49 PEP *n*= 51 IS	^R^*Pleasant Events Program* (Behavioral Activation) or time-equivalent Information-Support (IS) control^*^	Four 1-hr in-home therapy sessions (4 wks), followed by 2 weekly phone sessions (15 min-1 hr, caregiver directed time)	IL-6, D-dimer	baseline, post-intervention, 1 yr follow-up	No group differences in d-dimer; IL-6 reduced significantly more in the PEP participants compared to IS, at end of tx, but not 1 yr follow-up.
**von Känel et al. (2020)**	123 (77%); *n*= 60 PEP *n*= 63 IS	“Pleasant Events Program” (behavioral activation; PEP) or Information Support (IS)^*^	Six 60-min face-to-face therapy sessions over 12 wks	IL-6,CRP, TNF-α, D-dimer, PAI-1, VWF	baseline, post-tx (12 wks after baseline)	There were no significant time × treatment interactions for any biomarker; no significant time main effects, suggesting that biomarker levels did not change from pre- to post-treatment. However, several indicators of caregiver burden/distress and resources were predictive of a decrease in biomarker levels. Indicators of caregiving stress moderated tx effects on biomarkers.
**de Dios-Rodriguez et al. (2023)**	176 (72%); *n*=98 intervention *n*=78 control	Primary Care Physical Activity Program or Control group^†^	15-20 min interview, 30 mins moderate activity 5 days/wk or 20 min of vigorous activity 3 days/wk; 15 min interviews every 3 wks for 3 mos	Blood Pressure	pre-intervention, 6 mo follow-up after initial visit (intervention lasting for 3 of those mos)	The Primary Care Physical Activity intervention resulted in improved systolic blood pressure in comparison to the control.
**Grant et al. (2003)**	55 (61%); *n*=32 Respite *n*=23 Non-Respite	^R^In-home respite or non-respite / passive observation^†^	10 days of in-home help (max 6 hrs/day) within 2 wks	Blood pressure reactivity, heart rate reactivity, norepinephrine (NE) & Epinephrine (E) reactivity to acute stress	Baseline (pre-intervention), between days 7-9 of intervention, 1 mo follow-up (1 mo post-intervention). Blood samples for epinephrine and norepinephrine were taken at each visit at the end of a baseline and following two acute stressors	No tx effect of respite on changes in NE, blood pressure or heart rate from pre- to post. Vulnerable caregivers who received respite showed decreases in E from pre-respite to post-respite, whereas vulnerable caregivers who did not receive respite showed rises in E from pre-respite to post-respite.
**Williams et al. (2010)**	116 (77%); *n*= 59 VCS *n*= 57 Control	Video-based Coping Skills (VCS) and telephone coaching or Waitlist^†^	10 video modules 7-10 mins each (Ps viewed 2 modules per week + exercises & homework in a workbook); telephone coach calls 1x / wk for 5 wks	Salivary Cortisol, Blood Pressure reactivity	Baseline, 7 wks, 3 mos, 6 mos after visit 1. Salivary cortisol was assessed at the beginning of a laboratory visit, and after completing an acute psychological stressor. Salivary cortisol was also assessed the following day upon awakening, 30 min after awakening and after 6pm	DBP was lower in VCS than control across follow-up visits; DBP and SBP showed a larger decline in 6 mo follow-up period, compared to control; improvements in BP sustained to 6 mos. Neither mean levels of cortisol across visits nor the changes across the day or pre/post-stress differed significantly between the VCS and Waitlist groups.

*Notes.*

* Indicates an active control group,

† Indicates an inactive control group,

R Indicates random assignment to trial arms,

SR Indicates semi-random assignment to trial arms, IL-6 = interleukin 6, TNFα = tumor necrosis factor alpha, CRP = c reactive protein, NK cell= Natural Killer cell, PAI-1=Plasminogen activator inhibitor, VWF=von Willebrand factor, tx= treatment, hr(s)= hour(s) mo(s)= month(s), wk(s)= week(s), min= minutes.

Of the studies that analyzed salivary cortisol, six captured the diurnal rhythm of cortisol secretion at multiple timepoints during their intervention studies, and two studies examined morning cortisol awakening response (i.e., the change in cortisol from the first to the second saliva sample collected 30 minutes after awakening). Williams and colleagues (2010) collected cortisol data across four study visits (at baseline, seven-week follow-up, three-month follow-up, and six-month follow-up). Researchers collected a salivary cortisol sample at the beginning of each study visit, at the conclusion of an experimental stressor during each visit (i.e., continuous 4-5-minute speech about a caregiving experience that they found difficult to manage), and three additional samples on the day after each visit: upon waking, 30 minutes after waking, and the last sample after 6:00 pm. There were no significant differences in cortisol levels (neither mean levels across visits, diurnal cortisol rhythm, nor stress responses) between those assigned to a 5-week video-based coping skills training intervention with telephone coaching, and a waitlist control group [34].

Oken and colleagues (2010) randomly assigned participants to one of three conditions: Mindfulness-Based Cognitive Therapy, dementia education [active control] condition, or a respite-only [pragmatic control] condition. Those in the mindfulness meditation intervention (adapted from Mindfulness-Based Cognitive Therapy) engaged in a 90-minute Mindfulness-Based Cognitive Therapy session once per week for 6 weeks and implemented mindfulness skills at home. The second active intervention was a dementia education class (adapted from Powerful Tools for Caregivers), and a respite-only control that matched the duration of the two other interventions. At baseline and after the interventions (i.e., within 3 weeks pre- and post-intervention), researchers collected salivary cortisol 3 times during the assessment day; within 5 minutes after awakening, 30 minutes later (before eating), and at bedtime, approximately 10-11 pm. While participants in both the experimental groups reported significantly less caregiver stress post-intervention, there were no significant group differences in cortisol levels [35].

Holland and colleagues (2011) tested the effects of a cognitive-behavior-based psychoeducational intervention among caregivers of family members with ADRD. Each intervention treatment group attended 2-hour weekly sessions ranging between 13-16 weeks. Participants collected their saliva samples to measure salivary cortisol at wake, 5:00 pm, and 9:00 pm across two consecutive days at baseline and post-treatment. Only the intensity of caregiving (i.e., longer hours of care and living with care recipient) was significantly associated with baseline cortisol levels. Here, high-intensity caregivers had a more dysregulated diurnal cortisol pattern, with lower levels at wake and a flatter slope across the day, compared to low-intensity caregivers. There were no significant differences in post-treatment cortisol between conditions, suggesting that overall, engaging in the Coping with Caregiving intervention did not significantly impact diurnal cortisol rhythm compared to the control group (i.e., telephone support control). However, a significant intervention condition × intensity of caregiving interaction effect on both post-treatment awakening cortisol and diurnal cortisol slopes was reported. High-intensity caregivers in the intervention group displayed higher awakening cortisol and steeper diurnal cortisol slopes in comparison to the high-intensity caregivers in the control group. Thus, after the Coping with Caregiving intervention, high intensity caregivers no longer appeared to be particularly ‘at risk’ for having a dysregulated cortisol pattern. Therefore, it may be that the Coping with Caregiving intervention has the potential to improve health among high-intensity caregivers, but not among all caregivers, in general [36].

Danucalov and colleagues (2013) examined salivary cortisol levels before and after an 8-week yoga and compassion meditation program, compared to an untreated control group. The intervention sessions were a mix of in-person and video formats that lasted 1 hour and 15 minutes, three times a week, for two months. Four saliva samples were collected prior to the intervention; 2 samples on two consecutive days. These samples were taken immediately after waking up, while still in the dark, and 30 minutes later, in the natural light, due to the potential effects of light on cortisol concentration. Similarly, following the 8-week intervention, 4 samples were again collected using a similar procedure (i.e., 2 samples on two consecutive days immediately and 30 minutes after waking). There was a significant time × group interactional effect. Here, the treatment group had a significant reduction in average cortisol levels collected on day 2, as well as a significant reduction in the average cortisol levels across the two days. No significant changes were noted amongst the control group [37].

Aboulafia-Brakha and colleagues (2014) compared an 8-week, weekly cognitive-behavioral group therapy (CBT) intervention to a psychoeducation group program, in their ability to impact cortisol secretion. Samples were collected immediately after waking up (when still in bed), 30 minutes after waking up, eight hours after the first sample and immediately before going to sleep. The researchers reported a significant time (pre- vs. post-intervention) × group (CBT vs. psychoeducational) interactional effect on diurnal cortisol secretion. While the CBT group displayed a significant decrease in daily salivary cortisol from pre- to post-intervention, no significant change was observed amongst the psychoeducation group. Furthermore, a different diurnal cortisol pattern (i.e., high evening cortisol secretion) remained for the psychoeducation intervention participants post-intervention but disappeared for the cognitive-behavioral group therapy participants, whose final daily cortisol sample (immediately before going to sleep) was attenuated post-intervention [38].

Brown and colleagues (2016) conducted a study in which they compared an 8-week Mindfulness-based Stress Reduction (MBSR) intervention to a social support control group. Prior to the intervention, participants would attend an in-person session where they were provided with salivettes and instructions to collect saliva samples at baseline, post-intervention (8 weeks), and 3-months following the intervention. Samples were taken before rising in the morning, 45 min after awakening, 2.5 h after awakening, 8 h after awakening, 12 h after awakening, and at bedtime, at each of the 3 study phases. Neither treatment condition showed significant changes in diurnal cortisol trajectories over time (from pre- to post-intervention) [39].

In a randomized controlled trial (RCT), Prick and colleagues (2017) tested the effects of a multi-component dyadic intervention on 111 informal caregiving dyads by measuring caregiver salivary cortisol levels. Dyadic intervention participants completed eight home visits with a personal coach over three months. Visits included physical exercise and caregiving support (i.e., psycho-education, communication skills and pleasant activities training), whereas the control group received monthly information bulletins and phone calls from a coach [40]. Salivary cortisol measurements were taken directly after awakening and 30-minutes after awakening at baseline and 3 months post-baseline. No significant differences between the two groups were found from baseline to post-intervention for salivary cortisol at the time of morning awakening, or cortisol awakening responses [40].

Waelde and colleagues (2017) tested the Inner Resources for Stress mindfulness meditation and mantra training intervention throughout a period of 12 weeks (i.e., 10 sessions), relative to a psychoeducation and telephone support control condition for family dementia caregivers. Saliva samples were collected across two days (at awakening, 5 pm, and 9 pm) by participants at a baseline visit (1-week pre-treatment) and a follow-up visit (1-month post-treatment). Caregivers in the treatment group exhibited significantly improved (steeper) diurnal cortisol slopes, compared to those in the control condition [41].

### 3.1.2 Epinephrine and Norepinephrine

The catecholamines epinephrine and norepinephrine are stress hormones secreted in response to environmental stressors or emotional distress, also known as the “fight-or-flight” response. The measurement of these stress hormones allows for a standardized way of observing how the peripheral nervous system is activated in response to stress [42]. Circulating catecholamines (i.e., epinephrine, norepinephrine), are central to the functioning of the sympathetic-adrenal-medullary (SAM) axis. Following cognitive emotional responses to external stress, descending sympathetic activation via preganglionic and postganglionic nerve fibers stimulate the secretion of epinephrine and norepinephrine by the adrenal medulla into the blood stream. Subsequently, epinephrine and norepinephrine bind to adrenergic receptors in the cardiovascular system and influence heart rate and vascular contractility [43]. Thus, it has been posited that catecholamine activation within the SAM axis may promote adverse cardiovascular conditions including arterial inflammation, atherosclerosis, myocardial ischemia, and acute cardiovascular events [44]. In fact, elevated endogenous epinephrine and norepinephrine have been associated with an increased risk of developing hypertension [45]. Additionally, norepinephrine and epinephrine concentrations/reactions to psychological stress have both (respectively) been associated with increased resting blood pressure [59], as well as cardiovascular risk factors (e.g., Body mass index [BMI]) [46, 47].

The remaining two studies had samples measuring the stress hormones epinephrine and norepinephrine. Kim et al., (2011) tested 8 once-weekly support group sessions, compared to a control group that did not receive any intervention. One blood sample was taken in the morning between 9:30-10:30 am at both pre- and post-intervention. No significant changes in epinephrine from pre- to post-intervention were observed for either the experimental or control group. Similarly, there was no significant difference in the degree of change in epinephrine from pre- to post-intervention between groups. However, there was a significant treatment effect on norepinephrine, with those receiving the support intervention showing significantly less of an increase in norepinephrine in comparison to the control group [48].

Another study measured plasma epinephrine and norepinephrine in a treatment group receiving in-home respite care compared to no respite care [42]. Caregivers assigned to the treatment group were provided with 10 days of in-home help over a 2-week period. Epinephrine and norepinephrine were measured at baseline, before the 2-week in-home respite intervention, and at a one-month follow-up. Blood samples were taken following a 10-minute resting baseline, and after completing two stress tasks (i.e., 3 samples obtained during each visit). One of the stressors involved a rehearsed response to a shoplifting accusation, while another stressor was related to caregiving. At the follow-up, a slightly modified stressor was employed whereby participants were required to stand up for themselves against a disreputable automobile repair shop. There were no significant treatment main effects on epinephrine or norepinephrine observed between baseline and 1-month post-respite. However, there was a significant interaction effect with caregiver vulnerability. Those classified as vulnerable caregivers (i.e., providing more than 12 hours of caregiving and receiving in-home respite less than one per month) displayed a significant reduction in epinephrine from pre-to-post treatment in the respite intervention, while vulnerable caregivers in the control group displayed an increase in epinephrine. No significant intervention effects were observed for norepinephrine [42].

### 3.1.3 Summary of Cortisol, Epinephrine and Norepinephrine

In summary, of the ten studies examined, eight studies measured salivary cortisol pre- and post-intervention. Participants exhibited improved cortisol patterns in three of the eight in which researchers indexed cortisol pre-to-post-intervention [37, 38, 41]. The interventions most successful at improving diurnal cortisol rhythm included a yoga and compassion meditation intervention [37], a mindfulness meditation intervention [41], and a cognitive-behavioral group therapy intervention [38]. Yet, we are hesitant to conclude that these interventions alone are the reason for observed cortisol changes, given that researchers tested other interventions with similar components (e.g., mindfulness meditation), and did not find pre-to-post-intervention cortisol improvements [35, 39]. Only one of the eight studies investigated cortisol reactivity in response to an induced stressor and reported no significant treatment effects [34]. However, it is also important to note that while Holland and colleagues (2011) reported no significant treatment effects of a cognitive-behavioral based psychoeducational program, they did report a significant interaction between treatment group and caregiver intensity. High intensity caregivers (i.e., those putting in longer hours of care and living with the care recipient) displayed less dysregulated cortisol patterns (i.e., awakening cortisol and diurnal slopes) post-treatments in comparison to high intensity caregivers in the control group [36].

As seen in Table 1, the time points at which samples were collected varied across studies, with four studies requiring participants to collect salivary cortisol at three timepoints per day to capture cortisol diurnal rhythm [34-36, 41], one study collecting four samples, [38] and one study collecting six samples [39]. The final two studies asked participants to collect samples at waking and 30 minutes post-waking [37, 40]. One study asked participants to collect their waking sample in the dark, and their post-waking sample after being exposed to natural light [37]. When salivary cortisol was measured, participants were provided with instructions for the collection, storage, and transportation of the samples. While most studies employed analyses to examine diurnal cortisol slopes or cortisol changes across the day [36, 38, 39, 41], others examine treatment effects on single time cortisol samples [34, 35, 37, 40], averages of cortisol samples taken [37], or cortisol awakening responses [40]. Additionally, one study also examined change from pre- to post- cortisol measures in response to an acute stressor [34].

Along with the interventions ranging from 5 weeks to 16 weeks, studies varied in the timeframe of saliva collection in relation to the intervention cessation. While some did not specify the length between intervention cessation and post-treatment cortisol assessment, others reported assessing post-treatment cortisol within 3 weeks [35], at 1 month [41], and 2 months [36] after the intervention finished. Others provided the timeframe of cortisol assessment relative to pre-intervention (baseline measures). Here, post-intervention cortisol samples were taken 2 months after baseline (i.e., 2-month intervention; [37]) 8-weeks/3 months after baseline (i.e., 8-week intervention; [39]), 3 months post baseline (i.e., 3-month intervention; [40]), and seven weeks/three months/six months post-baseline (i.e., 5-week intervention; [34]).

Of the two studies that measured epinephrine and norepinephrine, one study examined a single blood sample taken at rest between the hours of 9:30 -10:30 am at pre- and post- intervention [48], while the other examined catecholamine responses to acute psychological stress utilizing 3 samples (i.e., end of baseline, post-stressor 1, post-stressor 2) [42]. While Kim et al. (2011) reports a treatment effect on norepinephrine, Grant et al. (2003) reported a treatment effect on catecholamines only amongst vulnerable caregivers [42, 48]. While Kim et al. (2011) did not report the specific timeframe of catecholamines in relation to the intervention, Grant et al. (2003) analyzed follow-up epinephrine and norepinephrine at 1 month following the intervention [42, 49].

### 3.2. Immunity

Higher levels of pro-inflammatory cytokines, including interleukin 6 (IL-6) and tumor necrosis factor-alpha (TNF-α) predict increased morbidity and mortality and decreased quality of life in older adults. Furthermore, spousal caregivers of people with ADRD demonstrate a four-fold increase in IL-6 levels, compared to matched controls [14, 16, 19]. Thus, chronic stress associated with caring for someone with ADRD negatively impacts health by dysregulating the immune system and increasing chronic inflammation, thereby prematurely aging the immune response [16, 21]. Based on our review, we found only four published (post-2001), peer-reviewed ADRD informal caregiving intervention trials which utilized immune biomarkers [35, 50-52]. Specifically, these studies utilized pro-inflammatory biomarkers (e.g., interleukin-6 [IL-6]); in fact, we found that these were the only immune biomarkers empirical researchers reported as part of ADRD informal caregiving intervention trials, post-2001.

### 3.2.1 Interleukin 6

Of the four studies that analyzed immune biomarkers, three reported interleukin 6 (IL-6) data. In a mindfulness meditation intervention study, Oken et al. (2010; see *Neuroendocrine Markers & Neurotransmitters* section above) assessed levels of IL-6, in saliva samples. Saliva samples were taken 3 times daily; 5 minutes after waking, 30 minutes later before eating, and at bedtime (10-11pm), at both pre- and post-intervention. No significant differences were found in inflammation between completers in either intervention group (i.e., Mindfulness-Based Cognitive Therapy, dementia education [active control] condition, or a respite-only [pragmatic control] condition), within three weeks of intervention conclusion [35].

Moore and colleagues (2013) examined whether a brief Behavioral Activation intervention would reduce biomarkers of cardiovascular disease, including IL-6 and D-dimer. Participants were randomly assigned to either the 6-week “Pleasant Events Program” (PEP) or a time-equivalent information-support control condition, each receiving four 1-hour in-home therapy sessions with varying material, followed by two weekly phone sessions (between 15 minutes - 1 hour). One blood sample was taken following a 20-minute resting period at baseline (i.e., pre-intervention), post-intervention, and at a 1-year follow-up. Participants in the treatment group demonstrated significantly lower IL-6 post-treatment, indicating lower Cardiovascular disease (CVD) risk, compared to those in the information-support condition; however, these significant reductions did not persist at a 1-year follow-up [52].

Similar to Moore and colleagues, von Känel (2020) and colleagues investigated whether a 12-week “Pleasant Events Program” (PEP) would result in a reduction of biomarkers, including IL-6, when compared to an active control group. One blood sample was collected by researchers at a pre-intervention baseline visit and post-intervention visit, both occurring at participants’ homes. Unlike the aforementioned study, there were no significant changes from pre- to post- in IL-6 levels for either the treatment or active control group [50].

### 3.2.2 D-dimer

In addition to analyzing IL-6, two of the previous studies also analyzed D-dimer, a protein that can be indicative of blood clotting [53] and is predictive of coronary heart disease [54]. Moore and colleagues (2013) collected one blood sample following a 20-minute resting period at baseline (i.e., pre-intervention), post-intervention and at a 1-year follow-up. There was no significant difference between the treatment and control groups on change in D-Dimer between baseline and either follow-up periods (i.e., post intervention and 1-year follow-up) [52]. Similarly, von Känel and colleagues (2020) collected one blood sample at both a pre-intervention baseline visit and at a post-intervention visit. There were no significant changes from pre- to post- in D-dimer levels for either the treatment or active control group [50].

### 3.2.3 Tumor necrosis factor alpha & C-reactive protein

Of the studies, two analyzed the immune biomarkers tumor necrosis factor (TNF-α) and C-reactive protein (CRP). Oken and colleagues (2010) collected saliva sampled 5 minutes after waking, 30 minutes later before eating, and at bedtime (10-11pm), at both pre- and post-intervention. There were no significant changes from pre- to post- in either TNF-α or CRP levels for the treatment or active control group [35]. Similarly, von Känel and colleagues (2020) collected one blood sample at both pre- and post-intervention and found that there was no significant difference between treatment and control groups on changes in TNF-α or CRP levels from pre- to post-intervention [50].

### 3.3.3 Plasminogen activator inhibitor-1 & von Willebrand factor

The biomarkers plasminogen activator inhibitor-1 (PAI-1) and von Willebrand factor (VWF) were analyzed in one study by von Känel and colleagues (2020), who collected one blood sample at both pre- and post-intervention. However, there were no significant changes from pre- to post- in either PAI-1 or VWF levels for the treatment or active control group [50]. However, it is important to note that von Känel and colleagues (2020) did note several significant interactional effects between pre-intervention measures of caregiver stress (i.e., negative affect, positive affect, and functional impairment of care recipient), and treatment effects on several assessed biomarkers [50].

### 3.3.4 T-cell proliferative response and Natural Killer (NK) Cell cytotoxicity

The fourth study by Garand and colleagues (2002) was the only to investigate T-cell proliferative response of peripheral blood mononuclear cells (PBMCs) to the mitogens Phytohemagglutinin (PHA) Sigma, Concanavalin A (ConA) Sigma, and Natural Killer (NK) Cell cytotoxicity. One blood sample (40 cubic centimeters) was taken at baseline, one-week post-intervention, and after 6 months of biweekly telephone support. To control diurnal variation, blood samples were obtained within a two-hour window. The T-cell proliferative response and NK Cell cytotoxicity was assessed after a six month Progressively Lowered Stress Threshold (PLST) psychoeducation telephone intervention, and compared to a control group engaging in a similar telephone interaction that provided only information and support [51]. Although the interaction between treatment group and time for immune outcomes were not significant (i.e., no significant differences in the degree of change over time between groups), there was a significant main effect of treatment on T-cell proliferation to PHA and ConA. Follow-up analyses revealed that at both one week following the in-home intervention, and after 6 months of telephone support, the treatment group displayed significantly stronger T-cell proliferation than the control group [51].

### 3.3.5 Summary of Immune biomarkers

In summary, only four ADRD informal caregiving intervention studies included immune outcomes (i.e., pro-inflammatory biomarkers IL-6, TNF-α, CRP, D-dimer, PAI-1, VWF, NK cell activity and T-cell proliferation to PHA & ConA), each yielding different results. Of the three studies analyzing IL-6, only one reported a significant treatment effect [52], while the remining two studies reported null-effects [35, 50]. Additionally, neither of the two studies which examined D-dimer reported significant treatment effects [50, 52]. Similarly, of the two studies which examined TNF-α and CRP, neither study reported significant differences between the treatment and control group at post-treatment/change in pre-to-post treatment [35, 50]. The one study that examined changes in PAI-1 and VWF from pre-to-post intervention reported no significant differences between treatment and control [50]. While the one study that examined T-cell proliferation and natural killer (NK) cell cytotoxicity did report between group differences for post treatment T-cell proliferation, there was no significant difference between groups in change of T-cell proliferation and NK cell cytotoxicity from pre-to-post intervention [51].

Across studies examining inflammatory biomarkers there was slight variability in the length of intervention, with interventions lasting 6 weeks [35, 52], 12 weeks [50], and 6 months [51]. Additionally, while some studies examined immune markers using blood samples [50-52], one study used saliva samples [35]. There was some variation between the end of the intervention and the collection of post-intervention immune samples, with samples collected immediately after the intervention [50, 51], within 3-weeks after the intervention [35], and immediately post treatment/at a 1-year follow-up [52].

### 3.4 Autonomic Psychophysiology Indices: Blood Pressure and Heart Rate

Williams et al. (2010; see *Neuroendocrine Markers & Neurotransmitters* section above) included blood pressure and heart rate reactivity measures in their controlled clinical intervention trial investigating whether video-based coping skills training with telephone coaching reduces psychosocial and biological markers of distress in informal ADRD caregivers. Heart rate and blood pressure were assessed every minute across rest (10 minutes), stress (i.e., recalling difficult caregiving experiences for 4-5 minutes), and recovery periods (5 minutes) across four study visits (at baseline, seven-week follow-up, three-month follow-up, and six-month follow-up). There was a significant group × time interaction for systolic blood pressure and diastolic blood pressure (i.e., average across baseline, stress, and recovery periods). Here, the treatment group had significantly lower blood pressure across the three follow-up visits and mounted a greater decrease in blood pressure at the final visit, six months post-baseline. In fact, the treatment group showed a decrease in systolic and diastolic blood pressure by 8 mmHg and 4 mmHg, respectively, by six months follow-up. However, there were no significant differences between the Video-based Coping Skills/telephone coaching and waitlist control on blood pressure or heart rate *reactivity* (i.e., the degree of change) to stress [34].

De Dios-Rodriguez and colleagues (2023) conducted a study observing both people with dementia and their family caregivers, when incorporating a program promoting physical activity (PEPAF) in primary care over the span of three months [55]. It was not reported when or how blood pressure was collected, though the provided table indicates that blood pressure was taken at baseline and six months post-baseline. In caregivers, the treatment condition produced a significant improvement in systolic blood pressure from baseline to a 6-month follow-up [55]. Grant and colleagues (2003; see *Neuroendocrine Markers & Neurotransmitters* section above) measured blood pressure reactivity and heart rate reactivity in a treatment group receiving in-home respite care compared to no respite care. Blood pressure and heart rate were continuously assessed using a blood pressure monitor on the participant’s finger during in-person visits before the 2-week in-home respite intervention and at a one-month follow-up. Blood pressure was assessed during a baseline period (10 minutes) and throughout two speech stress tasks (6 minutes each), at similar times of the day for each participant. There were no significant main or interaction effects of treatment or treatment × time interaction for diastolic blood pressure, systolic blood pressure, or heart rate [42].

### 3.4.1 Summary of Autonomic Psychophysiology Indices

Of the 3 studies examining autonomic psychophysiology indices, two studies reported significant treatment effects [34, 55], and one reported no significant intervention effect [42]. The durations of intervention were 2 weeks [42], 5 weeks [34], and 3 months [55]. While De Dios-Rodriguez and colleagues (2023) did not detail how blood pressure was assessed, both Grant and colleagues (2003) and Williams et al. (2010) included an acute stress testing protocol [34, 42, 55]. Grant and colleagues (2003) examined blood pressure during a 10-minute baseline and two 3-minute speech stressors (blood pressure and heart rate continuously measured every minute). Similarly, Williams et al. (2010) examined heart rate and blood pressure during a 10-minute baseline (assessed at 1-minute intervals), during a 4-5-minute speech task (5 measures at 1-minute intervals), and during a 5-minute recovery (5 measures). There was also slight variability between the end of the intervention and the assessment of post-intervention autonomic measures. Here, post-assessment measures were assessed at 1-month following the intervention [42], at 3 months following the intervention (i.e., 6 months post baseline assessment; [55]), and at seven weeks/three months/six months post baseline (i.e., 5 weeks intervention; [34]).

## 4. DISCUSSION

Informal caregivers of people with ADRD experience unique stressors, reduced quality of life [4, 5], and an increased risk for morbidity and mortality [4, 43, 56]. Over the last two decades, biobehavioral researchers began investigating immune, neuroendocrine, and autonomic physiological pathways, which may partially explain why informal caregivers of people with ADRD endure increased health risks. Understanding which interventions for those informally caregiving for someone with ADRD (e.g., therapeutic, psychoeducational, social support) may help alleviate the physiological and immune dysregulation associated with chronic stress, and in turn, reduce their disease risk, is imperative to mitigate the negative impacts of caregiving on caregiver health. We identified and reviewed 14 empirical studies, which tested a variety of caregiver interventions, to see whether participants showed improvement in stress-related biomarkers.

We found that the majority of ADRD informal caregiver intervention research studies utilizing biomarker(s) measured cortisol (i.e., 8 studies) a stress hormone and clinically relevant neuroendocrine marker. Overall, our review demonstrated mixed cortisol results. Participants exhibited improved cortisol patterns in one-third of the reviewed studies [37, 38, 41], and no improvements in two-thirds of the reviewed studies measuring cortisol pre-to-post-intervention [34-36, 39, 40]. There may be a variety of reasons for these mixed findings including a range of sample sizes (*N*s ranged from 27 to 176 participants; thus, influencing power; [38, 55]), variation in intervention components, intervention dosing (i.e., the amount of intervention needed to change the target outcome), and intervention duration. In addition, the studies varied in their design and measurement timepoints, both for cortisol assessments (mostly diurnal) and intervention follow-up time points. Further, studies differed in their inclusion/exclusion criteria, and many of the studies did not measure (or control for) certain physiological factors that may influence diurnal cortisol rhythm (e.g., menstruation, for cycling women; [57]). In a related vein, it is important for researchers to consider that caregivers of people with ADRD exhibit atypical cortisol slopes. As demonstrated in the Aboulafia-Brakha and colleagues (2014) study pre-intervention data, caregivers exhibited an atypical cortisol pattern with higher cortisol levels in the evening compared to the afternoon. This unusual cortisol increase may stem from more caregiving-related stress during evening rituals (e.g., mealtime, showering, helping to bed), and increased ADRD-related neuropsychiatric symptom expression (e.g., aggressiveness, hallucinations, delusions) in the evening [38]. Thus, appropriately analyzing diurnal cortisol data collected from caregivers of people with ADRD is very important; inappropriate analysis or conceptualizations may contribute to inaccurate conclusions. Researchers varied in their approach to analyzing the (mostly) diurnal cortisol data collected in the reviewed studies. Because in some cases, different analytic approaches may yield different results for the same data (i.e., area under the curve vs. repeated measures vs. mixed modeling), it is difficult to meaningfully compare cortisol results across studies. Lastly, we recommend that researchers consider intensity of caregiving as a possible moderating variable in future ADRD informal caregiving intervention studies measuring cortisol, given that Holland and colleagues (2011) found that those with higher intensity caregiving situations (i.e., longer hours of care and living with the care recipient) had less adaptive cortisol patterns (i.e., lower cortisol levels at wake and a flatter slope across the day), than those with lower intensity situations [36]. It could be that we are more likely to see interventions change diurnal cortisol rhythms among caregivers in high intensity caregiving situations because they have the flattest cortisol slopes (and thus, most dysregulated cortisol rhythm) to begin with. In fact, interactions between treatment and caregiving intensity/pre-intervention burden were noted in three studies [36, 42, 50], where the interventions were shown to be effective for these high-risk caregivers. Unfortunately, there may be a lack of representation of high intensity caregivers in most studies, given that these caregivers often have more barriers to research participation. Researchers who wish to investigate intensity of caregiving may have to tailor their recruitment and retention strategies to target high intensity caregivers to provide enough variability to detect intervention effects.

While both studies examining epinephrine and norepinephrine reported significant treatment effects, definitive conclusions pertaining to the effectiveness of interventions for these physiological parameters should be taken tentatively, based on the limited number of studies included [42, 48]. Kim et al. (2011) reported a treatment effect, with those receiving a support intervention showing less of an increase in norepinephrine in comparison to the control group. Grant et al. (2003) also reported a significant treatment effect on epinephrine (i.e., 10 days of respite), but only amongst vulnerable caregivers who provided care for more than 12 hours per day and received respite less than once per month over the past six months.

The four existing ADRD informal caregiving intervention studies, which included immune outcomes (i.e., pro-inflammatory biomarkers IL-6, TNF-α, CRP, NK cell activity, T-cell proliferation, D-dimer, PAI-I, VWF) yielded different results. These varying results may stem from several differences between the studies, including the intervention types and sample collection timing and method. Oken et al. (2010) found no evidence for Mindfulness-Based Cognitive Therapy reducing inflammation compared to both an active control group and a pragmatic control group; the active control group closely matched the time, intensity, and required participant engagement [35]. Similarly, von Känel et al. (2020) reported no significant treatment main effect of a Pleasant Events Program (versus an informational support group) on inflammatory biomarkers [50]. Moore and colleagues (2013) found that a behavioral activation intervention did significantly lower inflammation, compared to an active (information-support) control group. However, these reductions did not persist at a 1-year follow-up, suggesting that an additional dosing of intervention components (e.g., additional behavioral activation therapy sessions, additional phone calls, or booster sessions phone calls) may be needed during the post-treatment follow-up period to enhance the longevity of potential anti-inflammatory treatment effects [52]. Garand et al. (2002) reported a significant intervention effect on T-cell proliferation, whereby those receiving a lowering stress threshold psychoeducational intervention displayed stronger T-cell proliferation, in response to PHA and ConA, post-treatment in comparison to a group receiving matched support [51]. It is important to note that these studies varied in their collection methods of inflammatory biomarkers with one study employing saliva samples [35], and the remaining three studies using blood plasma samples [50-52]. Nevertheless, studying inflammatory biomarkers in ADRD informal caregiving intervention studies is extremely important, given that caring for someone with ADRD is associated with risk for immune dysregulation, thereby prematurely aging the immune response [16, 21]. Interventions offer one possible treatment modality, which may begin to reverse dysregulated immune responses associated with caregiving. However, more ADRD informal caregiving intervention trials must include immune parameters in their pre- and post-measures before we can identify and implement effective interventions.

Worth noting is a clear omission of autonomic physiological measures in most biobehavioral ADRD informal caregiving intervention studies. Only three studies measured cardiac-related or autonomic physiological outcomes (blood pressure, heart rate reactivity). Two of these studies found significant intervention effects [34, 55]. Williams and colleagues (2010) found that compared to controls, participants who completed video-based coping skills training showed greater improvements in average systolic and diastolic blood pressure than waitlist participants. In contrast, De Dios-Rodriguez and colleagues (2023) reported an improvement in systolic blood pressure amongst caregivers receiving a physical activity intervention in comparison with the control group [55]. However no between group differences (respite vs. no respite) in changes in blood pressure or heart rate were found by Grant et al. (2003). While blood pressure is an indicator of a person’s general heart health, other psychophysiological parameters such as vagal tone (indexed by high-frequency heart rate variability), capture one’s psychophysiology in a more nuanced way.

Vagal tone, or the activity of the vagus nerve, plays a vital role in physiological and behavioral reactivity [58]. The vagus nerve consists of sensory and motor fibers that operate between brain structures and visceral organs, establishing a face-heart connection, and impacts physiological regulation and the parasympathetic nervous system's influence on the heart [59-61]. The vagus nerve acts as a "brake," exerting an inhibitory influence on the sympathetic nervous system [60]. Vagal tone promotes health, in part, by fostering rapid, incremental changes in cardiac output to meet environmental demands [62]. Specifically, higher cardiac vagal tone is associated with decreased rates of physical illness among adults [63], while lower vagal tone is associated with increased respiratory problems, digestive issues, and cardiovascular disease [59, 64, 65]. Including psychophysiological measures, such as indexes of vagal tone, in future ADRD caregiving intervention studies may be key to understanding not only the secondary effects of stress, but also the flexibility of caregivers’ heart and emotional subsystems in responding to the demands of caregiving.

### 4.1 Implications and Directions for Future Research

There were substantial variations across studies pertaining to the intervention type, intervention duration, the number/timing of biomarker sampling, methods of biomarker collection, and follow-up assessment periods. Moreover, there was an insufficient number of studies to draw definitive conclusions pertaining to the utility of biobehavioral interventions for improving biomarkers amongst ADRD caregivers. Specifically, only eight studies examined cortisol, two examined epinephrine/norepinephrine, four examined immune biomarkers, and three examined autonomic functioning. Consequently, the current review identifies a pressing need for additional research, incorporating a broader array of biomarkers.

The intervention duration required to improve biomarkers amongst ADRD caregivers is unclear, with interventions ranging from 2 weeks to 6 months across studies. Therefore, we recommend that researchers continuously collect biomarker data throughout the intervention phase to identify the required “dosage” of a given intervention needed to improve the target outcome. Moreover, Moore and colleagues (2013) reported that the intervention effects did not persist at a second follow-up, 1-year later. Thus, future research should continue to collect biomarker data beyond the initial follow-up period to identify if, and when, caregivers require additional dosing of intervention components. Additionally, three studies accentuated the importance of considering caregiving intensity/pre-intervention burden when examining the effect of biobehavioral interventions on biomarkers; with treatment effects particularly observed amongst caregivers with greater caregiver burden/intensity [36, 42, 50]. While speculative, it may be that caregivers with substantially increased burden/intensity are at the greatest need of, and therefore receive greater benefits from biobehavioral interventions. Consequently, we suggest for future research to target “high risk” caregivers that report increased distress and caregiver burden, and/or to examine the potential moderating influence of self-reported caregiver burden on the effects of biobehavioral interventions on physiological parameters. These findings may also have implications for clinical practice and indicate that interventions may be most effective amongst caregivers who experience elevated burden and distress.

### 4.2 Summary and Conclusion

This systematic review article is the first to specifically examine ADRD informal caregiving intervention studies employing biomarkers. Interventions offer one possible treatment modality, which may begin to reverse premature aging associated with caregiving. We identified and synthesized 14 studies examining the effects of biobehavioral interventions on biomarkers amongst ADRD informal caregivers. These studies examined neuroendocrine and neurotransmitter, immune, cardiac and/or autonomic physiological indices. Findings were mixed across studies which may, in part, be due to differences in the types of interventions tested, as well as differing biomarker measurement, methodology, and analysis. Additionally, there was an insufficient number of studies to draw definitive conclusions pertaining to the utility of biobehavioral interventions for improving biomarkers amongst ADRD caregivers. Consequently, the present review accentuates the urgent need for further investigation of the effects of biobehavioral interventions for ADRD caregivers, encompassing a more diverse range of biomarkers.
